# The effect of the “Oral-Gut” axis on periodontitis in inflammatory bowel disease: A review of microbe and immune mechanism associations

**DOI:** 10.3389/fcimb.2023.1132420

**Published:** 2023-02-27

**Authors:** Tianyu Zhou, Wenzhou Xu, Qiqi Wang, Cong Jiang, Hongyan Li, Yang Chao, Yue Sun, Lan A

**Affiliations:** ^1^ Department of Oral Implantology, School and Hospital of Stomatology, Jilin University, Changchun, China; ^2^ Department of Periodontology, School and Hospital of Stomatology, Jilin University, Changchun, China; ^3^ Jilin Provincial Key Laboratory of Sciences and Technology for Stomatology Nanoengineering, Changchun, China; ^4^ Department of Gastroendoscopy, China-Japan Union Hospital of Jilin University, Changchun, China

**Keywords:** periodontitis, IBD, microbiology, immunity, oral-gut axis

## Abstract

Periodontitis and inflammatory bowel diseases (IBD) are inflammatory diseases of the gastrointestinal tract that share common features of microbial-induced ecological dysregulation and host immune inflammatory response. The close relationship between periodontitis and IBD is characterized by a higher prevalence of IBD in patients with periodontitis and a higher prevalence and severity of periodontitis in patients with IBD, indicating that periodontitis and IBD are different from the traditional independent diseases and form an “Oral-Gut” axis between the two, which affect each other and thus form a vicious circle. However, the specific mechanisms leading to the association between the two are not fully understood. In this article, we describe the interconnection between periodontitis and IBD in terms of microbial pathogenesis and immune dysregulation, including the ectopic colonization of the gut by pathogenic bacteria associated with periodontitis that promotes inflammation in the gut by activating the host immune response, and the alteration of the oral microbiota due to IBD that affects the periodontal inflammatory response. Among the microbial factors, pathogenic bacteria such as *Klebsiella*, *Porphyromonas gingivalis* and *Fusobacterium nucleatum* may act as the microbial bridge between periodontitis and IBD, while among the immune mechanisms, Th17 cell responses and the secreted pro-inflammatory factors IL-1β, IL-6 and TNF-α play a key role in the development of both diseases. This suggests that in future studies, we can look for targets in the “Oral-Gut” axis to control and intervene in periodontal inflammation by regulating periodontal or intestinal flora through immunological methods.

## 1 Introduction

Periodontitis is one of the most common oral diseases and a major cause of tooth loss in adults. Periodontitis is an infectious disease caused by plaque biofilm that leads to the destruction of tooth-supporting tissues, with clinical symptoms including attachment loss, periodontal pocket formation, and alveolar bone resorption, which, if left untreated, can gradually lead to tooth loosening or even tooth loss (Petersen and Ogawa, 2012; Kinane et al., 2017; Van der Velden, 2017). There is a growing body of research showing that the health of oral tissues interacts with systemic health (Taylor et al., 2000; Genco and Sanz, 2020; Kapila, 2021; Larvin et al., 2022; Teles et al., 2022). Notably, periodontitis is closely associated with several systemic diseases, including atherosclerotic cardiovascular disease (Beck et al., 2001; Van Dyke et al., 2021; Isola et al., 2022), diabetes (Chapple et al., 2013; Genco and Borgnakke, 2020; Nibali et al., 2022), adverse pregnancy outcomes such as miscarriage and preterm delivery (Canakci et al., 2004; Sanz et al., 2013; Miranda-Rius et al., 2021), rheumatoid arthritis(Bartold and Lopez‐Oliva, 2020; Möller et al., 2020; González‐Febles and Sanz, 2021), Alzheimer’s disease (Pizza, 2019; Kamer et al., 2020; Torrealba-García et al., 2021) and inflammatory bowel disease (Cai et al., 2021; Imai et al., 2021; Zhang et al., 2021a; Baima et al., 2022).

The oral cavity is a complex ecosystem in which microorganisms accumulate and multiply, forming dental plaque (Listgarten, 1994; Lourenço et al., 2014). Plaque is the basis for the survival, metabolism and pathogenesis of oral bacterial, and it can serve as an arsenal for bacterial pathogenesis, producing antigens that invade the deep periodontal gingival mucosa and interfere with the host immune defense system, thereby exacerbating the inflammatory response (Murakami et al., 2018; Valm, 2019; Jakubovics et al., 2021). The flora composition and oral ecology of patients with periodontitis becomes more complex, as Socransky et al. suggested that periodontitis is caused by specific pathogens such as “red complex” bacteria (*Porphyromonas gingivalis*, *Treponema denticola* and *Tannerella forsythia*)(Socransky et al., 1998), with Hajishengallis’ in-depth study of its pathogenesis revealing that the etiology of periodontitis is a synergistic effect of a dysregulated microbial community (Hajishengallis and Lamont, 2012), that is, bacteria invade the host tissue and disrupt its immune protective mechanisms, causing local periodontal tissue damage, while facilitating the enrichment of other anaerobic bacteria and increasing the pathogenicity of the entire microbial community, resulting in an inflammatory response.

IBD is a non-specific chronic inflammatory disease of the intestine that has received a lot of attention from scholars because of its high incidence (Windsor and Kaplan, 2019; Agrawal and Jess, 2022; Upadhyay et al., 2022), mainly including ulcerative colitis (UC) and Crohn’s disease (CD). UC mainly damages the colon and rectum, while CD can damage any part of the gastrointestinal tract from the mouth to the anus, with the end of the small intestine and the colon being most commonly affected (Guan, 2019). The main clinical manifestations of IBD are abdominal pain, diarrhea, bloody stools and weight loss (She et al., 2020). Approximately more than 40% of patients will present with extra-intestinal manifestations, such as oral, ocular, cutaneous, hepatobiliary, urinary and neurological lesions may be associated (Jose et al., 2009; Lankarani, 2013; Taleban et al., 2015; Hedin et al., 2018; Marotto et al., 2020). Oral lesions are the most common extraintestinal manifestation in patients with IBD, which can appear as the first symptom and are closely related to disease activity, mainly in the form of recurrent oral aphthae (Sircus et al., 1957), granulomatous inflammation (Dudeney, 1969)and periodontitis (Byrd and Gulati, 2021). In addition, IBD is associated with environmental factors, genetic susceptibility, gut microbiota and host immune response (Ananthakrishnan et al., 2017; Khalili et al., 2018; Sugihara et al., 2019; Amoroso et al., 2020; Agrawal et al., 2022), among which gut microbes are crucial for the development of IBD (Ni et al., 2017; Schirmer et al., 2019). Schreiber et al. found that bacterial diversity in the intestine of IBD patients was reduced, with CD patients having a reduced number of *Firmicutes* and an increased abundance of *Proteobacteria* and *Bacteroidetes*, while UC patients had a relatively mild degree of intestinal microbial dysbiosis, but the exact pathogenesis of IBD is still unclear (Ott, 2004).

**Figure 1:**
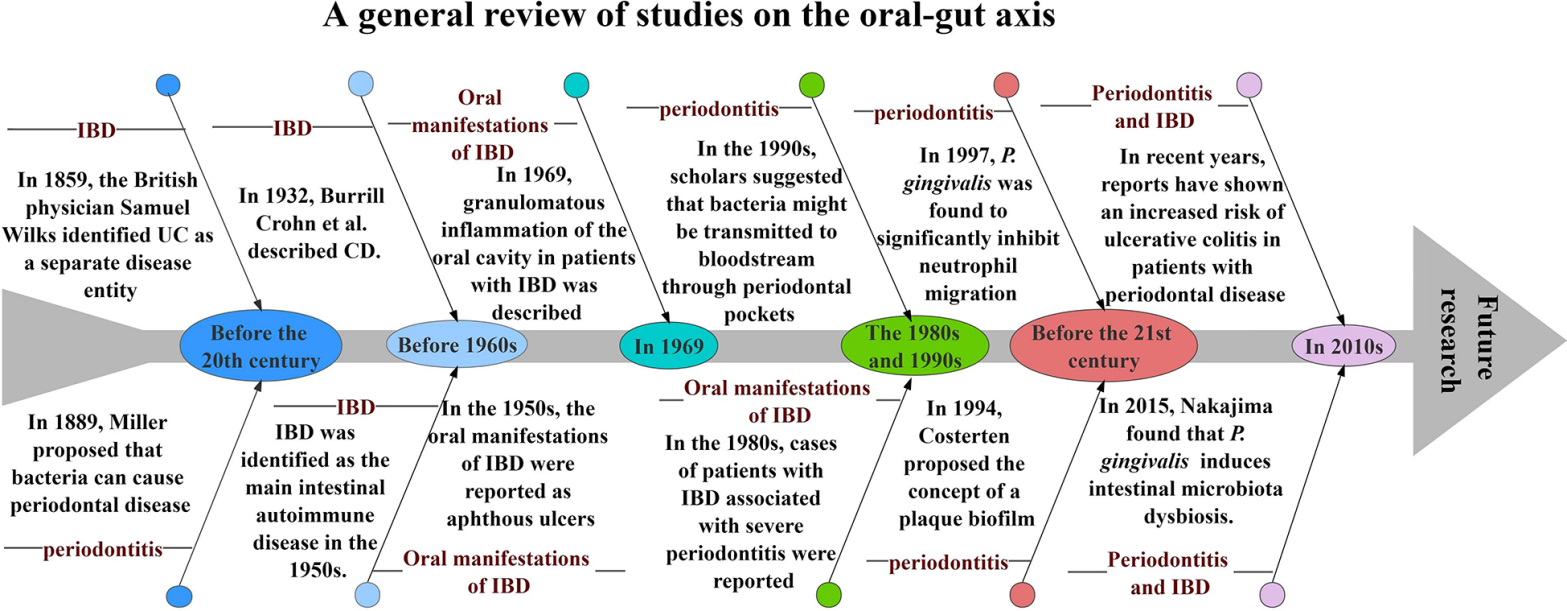
A general review of studies on the oral-gut axis.

Periodontitis, one of the most common diseases of the oral cavity, has been studied by countless scholars over the years. In 1889, Miller first linked the theory of bacteria to oral disease, clearly implying that bacteria play a role in the late stages of periodontal pathology (LÖE, 1993); in 1994, Socransky et al. classified microorganisms in subgingival plaque into five complexes according to their aggregation and pathogenicity (Socransky et al., 1998). Similar to periodontitis, the signs and symptoms of IBD have been mentioned throughout human history. In 1859, the British physician Samuel Wilks identified UC as a separate disease; in 1932, Burrill Crohn et al. described inflammation of the terminal ileum in an article, a disease that came to be known as CD; in the 1950s, it was noted that symptoms in patients with CD and UC responded to corticosteroids and IBD was identified as a major intestinal autoimmune disease (Malik, 2015). The oral manifestations of IBD were first reported in the 1950s, initially focusing on aphthous ulcers(Sircus et al., 1957); in 1969, Dudeney et al. described granulomatous inflammation of the oral cavity in patients with IBD(Dudeney, 1969); in the 1980s, cases of severe periodontitis in patients with IBD were reported(Engel et al., 1988). Periodontitis may lead to low-grade systemic inflammation and its association with other chronic inflammatory diseases has been studied for many years. In the 1990s, scholars suggested that oral bacteria may spread into the bloodstream through the ulcerated epithelium in periodontal pockets and cause transient bacteremia (Asikainen and Alaluusua, 1993); in 1997, Madianos et al. found that *Porphyromonas gingivalis*(*P. gingivalis*) significantly inhibited neutrophil migration induced by stimuli such as *Escherichia coli*(Madianos et al., 1997); in 2015, Nakajima et al. found that *P. gingivalis* causes changes in intestinal bacterial composition and induces intestinal microbiota dysbiosis and impaired barrier function(Nakajima et al., 2015); in recent years, scholars have reported a significantly increased risk of ulcerative colitis in patients with periodontal disease(Lin et al., 2018). All these studies and reports provide favorable evidence for the existence of an “oral-gut” axis in periodontitis and IBD (**Figure 1**).

The oral cavity and the intestine are the starting and ending points of microbial aggregation in the digestive tract, housing unique microbial groups associated with human health and disease (Lynch and Pedersen, 2016), and both share some common flora, including *Streptococcus*, *Bacteroides*, and *Prevotella* (Byrd and Gulati, 2021). Most studies have shown that microorganisms in the oral cavity can be transmitted to the intestine through the digestive tract (Lira-Junior and Figueredo, 2016; Atarashi et al., 2017; Schmidt et al., 2019; Kitamoto et al., 2020a; Byrd and Gulati, 2021). Periodontal pathogenic bacteria can ectopically colonize the gut from the oral cavity, leading to disruption of the intestinal microbiota and inducing an intestinal immune response, thereby exacerbating intestinal inflammation (Atarashi et al., 2017), suggesting a unique association between periodontitis and IBD.

At the same time, harmonious intestinal flora facilitates the control of periodontitis, and the administration of intestinal probiotics with inflammatory regulatory properties is considered one of several new approaches to address bacterial imbalances in periodontitis and prevent bone loss. Several clinical studies have shown that adjuvant oral administration of the intestinal probiotic (*Lactobacillus reuteri*) after basic periodontal treatment significantly improves periodontal clinical signs (Ikram et al., 2018) and that gavage of the probiotic significantly reduces the number of osteoclasts in the alveolar bone of mice compared with oral inoculation of the probiotic (Gatej et al., 2017). Treatment of IBD is closely related to periodontitis healing and prevention (Newman and Kamada, 2022). Probiotics such as *Lactobacillus* have beneficial activity for the treatment of gastrointestinal diseases such as IBD, and related longitudinal studies have shown that gavage of probiotics before the formation of periodontitis in mice resulted in a significant increase in β-defensin levels in intestinal and gingival tissues, significantly reduce the levels of inflammatory factors in gingival tissues during periodontitis while modulating intestinal barrier function and improving intestinal inflammation, and alleviate alveolar bone resorption and periodontal membrane destruction (Kobayashi et al., 2017). In addition, altering the progression of chronic systemic inflammatory disease through dietary control may have similarities to improving chronic adult periodontitis (Dawson et al., 2014). Studies have pointed out that certain therapeutic agents of CD, such as steroids, may be able to prevent periodontitis (Chi et al., 2018). Currently, psoralen, vitamin D, and other substances have been shown to achieve the treatment of periodontitis by repairing the barrier of intestinal inflammation damage (Jagelavičienė et al., 2018; Machado et al., 2020; Liu et al., 2021a). Moreover, Zhang et al. proved that with the improvement of periodontitis, IBD was also significantly relieved after exosome treatment (Zhang et al., 2021a). All of the above related longitudinal studies suggest a strong bidirectional association between periodontitis and IBD, with the treatment of one disease having a beneficial effect on the other, which inspires that future studies could use a drug to treat intestinal inflammation by improving the intestinal flora while having a beneficial effect on the prevention and control of periodontitis.

In terms of immune mechanisms, the digestive tract is the main site of communication between the internal and external environment and a key barrier against pathogen invasion, while its defense against pathogen invasion includes both intrinsic and specific immunity, of which the main one is the immune defense involving T lymphocytes. CD4^+^ T cells are the main cell population mediating various host protective and homeostatic responses and contain many subpopulations (e.g., helper T cells, Th1, Th2, Th17 and regulatory T cells, Treg) (Shale et al., 2013; Jaeger et al., 2021; Hong et al., 2022; Yang et al., 2022), of which two CD4^+^ T lymphocyte populations synergistically regulate adaptive immunity in the intestinal mucosa: peripherally induced regulatory T cells (pTreg cells) and CD8αα-expressing intraepithelial lymphocytes (CD4IELs) (Shale et al., 2013). However, the exact mechanism of how CD4^+^ T cells recognize colony antigens and differentiate into CD4IELs remains unknown. According to a recent study by Bousbaine’s team published in *Science* in 2022, β-hex-specific T cells induced by intestinal commensal antigens can drive differentiation of CD4IELs and regulate intestinal inflammation by expressing interleukin (IL)-10 (Bousbaine et al., 2022). This finding suggests us whether periodontal inflammation can be controlled and intervened by modulating oral or gut microbiota through immune means. Therefore, in this paper, we discuss the association between periodontitis and IBD from both microbial and immune mechanisms, which has important implications for the pathogenesis and treatment of periodontitis and IBD.

## 2 Correlations between periodontitis and IBD

### 2.1 Prevalence and comorbidity

Periodontitis as a global public health problem has received wide attention from scholars (Luo et al., 2021; Stødle et al., 2021; Alawaji et al., 2022; Ju et al., 2022; Wu et al., 2022). TaeHyun Kwon et al. found that about 42% of adults in the United States had periodontitis, and 7.8% of them had severe periodontitis (Kwon et al., 2020). In China, the prevalence of periodontitis in adults is as high as 80% to 90%, with chronic periodontitis accounting for 60% to 70% of the prevalence (Meng, 2007). An epidemiological study by Frencken et al. showed that the prevalence of severe periodontitis in the global population remained at about 11.2%, and the prevalence of severe periodontitis increased with age, with a sharp increase in the 30-40 years of age interval. It reaches a peak at the age of 40 years and remains stable thereafter (Frencken et al., 2017).

As an immune-mediated chronic inflammatory disease, the incidence and prevalence of IBD have continued to increase worldwide over time, indicating that it has become a global disease (Ye et al., 2019; Alatab et al., 2020; Kaplan and Windsor, 2020; Zhao et al., 2021). The oral cavity is one of the most vulnerable areas for extraintestinal manifestations of IBD, and numerous studies have found that patients with IBD have a higher risk of periodontitis than those without IBD (Papageorgiou et al., 2017; She et al., 2020; Zhang et al., 2021c; Abrol et al., 2022). In addition, the severity and extent of periodontitis suffered by patients with IBD was found to be greater, with patients having significantly higher mean probing depth, plaque index, calculus index, sulcus bleeding index, and attachment loss than patients with general periodontitis (Zhang et al., 2020; Nijakowski et al., 2021; Domokos et al., 2022). It has also been confirmed that patients with periodontitis have a significantly higher risk of developing IBD than healthy controls without periodontitis (Lin et al., 2018; Kang et al., 2020; Madsen et al., 2022). All of the above studies suggest a close bidirectional association between periodontitis and IBD.

### 2.2 Microbial associations of periodontitis and IBD

A large number of microorganisms colonize the human oral cavity, including bacteria, fungi, viruses, protozoa, and mycoplasma (Wade, 2013; Mosaddad et al., 2019). Among them, bacterial communities can reach more than 1000 species, including *Actinomyces*, *Bacteroides*, *Firmicutes*, *Proteobacteria* and *Spirochete* (Dewhirst et al., 2010). In most cases, bacteria in the oral cavity maintain a relative balance between flora, as well as a dynamic balance between flora and host; however, when the normal flora loses its mutual control or the microorganism and the host lose their balance, it will cause the oral ecological environment to become dysfunctional and the pathogenic bacteria will escape or inhibit the host’s defense function, damaging the host tissue and eventually leading to the development of periodontitis (Darveau, 2010). Mike et al. found a significant increase in oral microbial diversity in patients with periodontitis by 16S rRNA sequencing, with a high concentration of Gram-negative bacteria in the subgingival flora, including the classical “red complex” bacteria (Curtis et al., 2020). Notably, *Porphyromonas gingivalis* (*P. gingivalis*), a Gram-negative, specialized anaerobic bacterium, is considered to be the main pathogen involved in the onset and progression of chronic periodontitis (Reyes, 2021), attaching to mucous membranes, periodontal pocket epithelium and other bacterial surfaces by a series of adhesion factors, including bacterial hairs, hemagglutinins and proteases (Lamont and Jenkinson, 2000) and can secrete large amounts of proteases (e.g., gingipains), endotoxins (e.g., lipopolysaccharide, LPS), acid and alkaline phosphatases, indoles, organic acids, and other virulence factors that destroy periodontal tissue by degrading host proteins and evading host immune defenses(Jia et al., 2019a; Xu et al., 2020), leading to clinical manifestations of edema, neutrophil infiltration, and hemorrhage (Travis et al., 1997). In addition, Settem et al. demonstrated that *Fusobacterium nucleatum* (*F. nucleatum*) and *Tannerella forsythia* cause destruction of alveolar bone by synergistically stimulating the immune response in periodontal tissues (Settem et al., 2012), thus demonstrating that the synergistic action of ecologically dysregulated microbial communities plays an important role in the progression of periodontitis. The microbial metabolites are necessary for periodontal pathogenic bacteria to maintain their growth, reproduction and pathogenesis. Compared to healthy subjects, patients with periodontitis had significant differences in the composition of salivary microbial metabolites with higher levels of serine, serotonin, 4-hydroxycinnamic acid, hydrocinnamic acid and isoleucine (Wei et al., 2022). Guan et al. noted that short chain fatty acids (SCFAs) are important pathogenic factors in periodontitis, and butyrate can damage the gingival epithelium by exerting destructive effects on intercellular junctions, leading to cell death (Guan et al., 2020).

The oral cavity and the intestine, as the two ends of the digestive tract, share similar microbial pathogenic mechanisms. When the intestinal microbial balance is disturbed, bacterial metabolites are altered and a large number of virulence factors are released, which can damage host tissues and eventually lead to an inflammatory response (Glassner et al., 2020; Lavelle and Sokol, 2020; Lee and Chang, 2020). The microbiota in the human gut consists mainly of *Firmicutes*, *Bacteroidetes*, *Proteobacteria* and *Actinobacteria*, with *Firmicutes* (49-76%) and *Bacteroidetes* (16-23%) predominating (Eckburg et al., 2005; Frank et al., 2007). Host tissues provide a nutrient-rich habitat for the gut flora, while the gut flora can provide short-chain fatty acids and essential vitamins to the host (Mukherjee et al., 2020), and the two complement each other in a harmonious symbiosis, a mutual relationship known as symbiosis; however, when suffering from IBD, the microbiota of the gut is altered, a change known as ecological dysbiosis (Matsuoka and Kanai, 2014). Frank et al. found that, in contrast to periodontitis, the diversity of the intestinal flora is reduced in patients with IBD, with a decrease in the number of *Firmicutes* and an increase in the number of *Proteobacteria* (Frank et al., 2007). Changes in the composition of the gut microbiota lead to altered bacterial metabolites, which may play a role in the pathogenesis of IBD. Bile salt hydrolase (BSH), a metabolite of intestinal bacteria (especially the thick-walled phylum), plays a key role in the modification of bile acids, which maintain the integrity of the intestinal epithelial barrier and improve intestinal inflammation through a negative feedback mechanism mediated by the farnesoid X receptor (FXR) (Gadaleta et al., 2011; Ding et al., 2015; Gonzalez et al., 2016). However, when compared to healthy individuals, the number of BSH in *Firmicutes* was significantly lower in the intestine of IBD patients, which led to a significant reduction in the anti-inflammatory effect of FXR (Ni et al., 2017). Additionally, intestinal microbiota metabolism can produce butyrate, an important energy source for intestinal epithelial cells, and studies have found that the number of butyrate-producing *Faecalibacterium prausnitzii* in the intestine of IBD patients is significantly reduced, while reduced butyrate levels induce an intestinal inflammatory response (Takaishi et al., 2008). Choline is an essential dietary nutrient for humans, and intestinal microorganisms (*Firmicutes*) play a key role in the degradation of choline to trimethylamine (TMA), which is further metabolized in the liver to trimethylamine N-oxide (TMAO), the level of which is associated with many adverse host pathologies, including IBD. It has been shown that TMAO levels are significantly elevated in mice fed a choline-rich diet, which leads to increased expression of pro-inflammatory cytokines, and that in colonic epithelial cells, TMAO triggers activation of inflammatory vesicles and production of reactive oxygen species in a dose- and time-dependent manner, thus playing a potential role in the pathogenesis of IBD(Hosseinkhani et al., 2021); in addition, recent studies have found elevated levels of circulating TMAO in patients with stage III-IV periodontitis(Zhou et al., 2021), suggesting a close relationship between intestinal flora metabolites and periodontitis. All of the above studies suggest that alterations in intestinal flora are closely related to the development of IBD.

It was found that microorganisms from the oral cavity can be detected at low levels in stool (Schmidt et al., 2019), suggesting that microorganisms from the oral cavity can spread to the intestine, which also allows pathogenic bacteria to ectopically colonize the intestine and consequently disrupt intestinal homeostasis and abnormally activate the intestinal immune system, thus affecting the development of IBD. Koji et al. suggest that the oral cavity can act as a potential reservoir for intestinal pathogens (Atarashi et al., 2017). For example, when periodontitis develops, *Klebsiella* and *Proteus* proliferate in the oral cavity and can migrate to ectopically colonize the lower gastrointestinal tract, thus causing activation of macrophage inflammatory vesicles, which may exacerbate intestinal inflammation (Kitamoto et al., 2020b). *Candida*, an important pathogenic fungus in the body, is usually found in the oral cavity and intestinal tract of normal people. Generally, small amounts of *Candida* do not cause disease, but when the body becomes ecologically imbalanced, it can multiply and cause disease. In recent years, numerous studies have found that *Candida* is closely associated with both periodontitis and IBD. Studies have shown that oral colonization by *Candida* is strongly associated with the severity of periodontitis. Candidalysin directly induces pro-inflammatory factors and NLRP3 inflammatory vesicles thereby promoting an inflammatory response, and inhibition of *Candida albicans* by antifungal agents is effective in reducing the severity of periodontitis in women (Ho et al., 2020). Hiengrach et al. found that oral *Candida* can promote the growth of intestinal bacteria thereby inducing intestinal ecological dysbiosis, which in turn promotes colonic inflammation (Hiengrach et al., 2020); *Candida albicans* colonize the gut significantly more frequently and with greater severity in patients with CD than in the healthy population (Standaert-Vitse et al., 2009), whereas reducing intestinal fungus reduces the severity of the disease. Furthermore, in the study by Li et al. oral *Candida* can colonize the gut through the digestive tract and disrupt the intestinal barrier by inducing the release of pro-inflammatory factors, while in the pathogenic mechanism of *Candida* candidalysin is a key virulence factor in promoting the inflammatory response in the gut (Li et al., 2022b). Oral *Candida* colonizes the intestine through the digestive tract and thus affects the metabolic and ecological balance of the flora. Another study found that after antibiotic treatment resulting in *Candida*-dependent oral and intestinal inflammation, SCFA supplementation promoted fungal clearance and restored Th17 and Treg cell SCFA by regulating T cell cytokines, thereby improving the inflammatory response (Bhaskaran et al., 2018). This close link between the oral and intestinal tracts is crucial to our study of the oral-gut axis, and it seems that inhibiting *Candida* colonization by modulating the intestinal flora as well as microbial metabolites, and thus improving periodontitis through the oral-gut axis may provide us with new therapeutic directions. Furthermore, *P. gingivalis* and *F. nucleatum*, as pathogenic bacteria of periodontitis, similarly affect the development of IBD. Lee et al. showed that the number of *P. gingivalis* in the stool of CD patients was significantly higher, and that *P. gingivalis* colonization of the intestine led to the loss of intestinal surface epithelium, destruction of crypt structures, and infiltration of inflammatory cells, ultimately exacerbating the manifestation of intestinal inflammation (Lee et al., 2022); Xiao et al. found that the establishment of a mouse periodontitis model by oral administration of *P. gingivalis* resulted in a significant increase in LPS levels and led to enhanced expression of flavin monooxygenase3 (FMO3) (FMOs promotes the conversion of TMA to TMAO) and plasma TMAO levels, which induced intestinal ecological dysregulation and inflammatory responses (Xiao et al., 2022); Jaclyn et al. found that *F. nucleatum* is present in the intestinal mucosa of IBD patients and that the invasive potential of *F. nucleatum* strains is positively correlated with the disease status of the host, thus inferring that *F. nucleatum* can be used as a potential biomarker for gastrointestinal diseases (Strauss et al., 2011). In addition to the microbes themselves, their metabolites can also enter the intestine *via* the oral-gut axis, thereby promoting inflammation in the gut. Wang et al. found that periodontitis upregulates the levels of the microbiota metabolite isoleucine (Ile) in saliva, and that Ile translocated through the digestive tract to the intestine and acetylated the NLRP3 protein through its metabolite AcCoA to aggravate colon inflammation in mice (Wang et al., 2022). Microbial metabolites are essential for the regulation of the ecology of the host digestive tract, which in turn suggests that they could be a common therapeutic target for periodontitis and IBD *via* the oral-gut axis.

Systemic inflammation in patients with IBD also has an impact on the composition of the oral microbiota, and Docktor et al. found an increase in the number of *Spirochaetes* and *Bacteroidetes* and a decrease in the number of *Firmicutes* and *Fusobacteria* in the oral cavity of patients with IBD(Docktor et al., 2012); Said et al. found a significant increase in *Prevotella* spp. in the salivary microbiota of patients with IBD by bacterial 16S rRNA sequencing, while *Streptococcus* spp., which are most abundant in saliva in healthy populations, were significantly reduced(Said et al., 2013). Another study found that IBD causes a disruption of the thick-walled bacterial gate in the patient’s oral cavity, the extent of which is closely related to the severity of the disease, and that this ecological dysregulation subsides over time after successful treatment of IBD (Elmaghrawy et al., 2022). These bacteria enriched in the oral cavity of IBD patients are in turn closely associated with periodontitis, and all of the above studies suggest that IBD causes the development of periodontitis by affecting oral ecological dysregulation.

**Figure 2:**
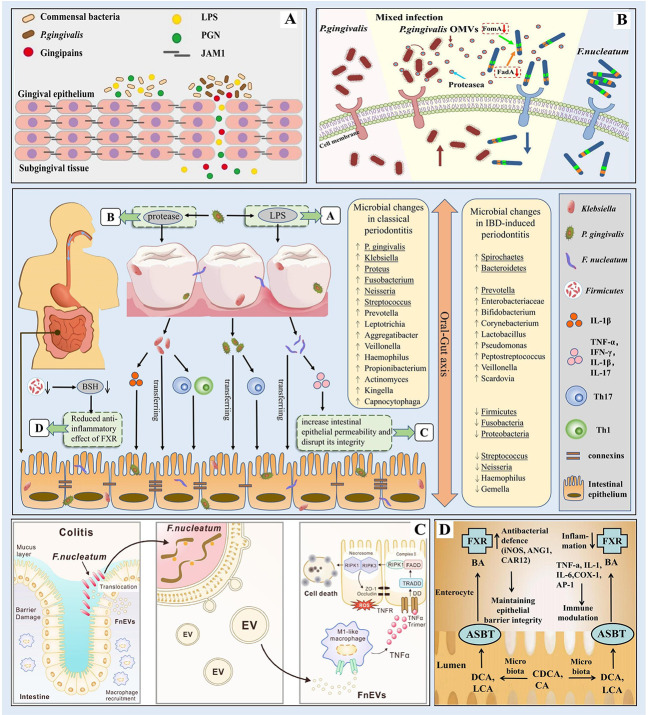
Microbial correlation between periodontitis and IBD (The bacteria underlined in the diagram are the ones mentioned in our paper). In periodontitis, the oral flora is altered and *P. gingivalis* evades host immune defense to destroy periodontal tissue by releasing virulence factors such as proteases and lipopolysaccharides; alterations in the composition of the intestinal microbiota leading to changes in bacterial metabolites such as BSH may play an important role in the pathogenesis of IBD; through the oral-gut axis, periodontal pathogenic bacteria such as *Klebsiella, P. gingivalis*, and *F nucleatum* can ectopically colonize the intestine and disrupt the intestinal barrier thus leading to intestinal ecological dysregulation and chronic inflammation. **(A)** *P. gingivalis* produces virulence factors such as LPS. Source (Takeuchi et al., 2019). **(B)** *P. gingivalis* produces virulence factors such as proteases. Source (Zhang et al., 2021d). **(C)** *F nucleatum* destroys the intestinal mucosa. Source (Liu et al., 2021b). **(D)** The role of FXR in IBD. Source (Ding et al., 2015).

Periodontitis-associated pathogenic bacteria can migrate to the intestine and interfere with intestinal barrier function, thus causing intestinal ecological dysregulation and chronic inflammation, while IBD can cause changes in oral microbial composition and lead to the proliferation of periodontitis-associated pathogenic bacteria, suggesting that oral flora disorders are closely related to intestinal flora abnormalities (**Figure 2**). This bidirectional association between the oral cavity and the intestine, i.e., the existence of an Oral-Gut axis, is of great significance and also suggests a possible common immunomodulatory mechanism between periodontitis and IBD.

### 2.3 Immune mechanism associations of periodontitis and IBD

Although pathogenic microorganisms can directly destroy host periodontal tissues by releasing virulence factors and metabolites, the host immune response plays an equally important role in the progression of periodontitis (Bosshardt, 2017; Bunte and Beikler, 2019; Pan et al., 2019). The host immune response to microorganisms can be divided into intrinsic immune responses and adaptive immune responses. The intrinsic immune system is the first line of defense against pathogen invasion and consists of different cells (neutrophils, macrophages, etc.) and factors (e.g. complement), among which neutrophils continuously recruit, migrate and infiltrate at the site of inflammation and secrete pro-inflammatory factors such as tumor necrosis factor (TNF)-α, IL-8 etc., but their phagocytic function is significantly reduced (Carneiro et al., 2012; Hajishengallis, 2019; Vitkov et al., 2021); macrophages tend to differentiate towards M1 (pro-inflammatory) in periodontitis and increase the secretion of pro-inflammatory mediators (e.g. interferon-γ, IFN-γ) as well as osteoclast activity (Almubarak et al., 2020; Sun et al., 2021; Yang et al., 2021). Moreover, T cell-mediated adaptive immune responses are crucial in the development of periodontitis, and upon activation through the T cell receptor (TCR), naive CD4^+^ T cells can differentiate into Th1, Th17, and Treg cells and participate in different types of immune responses (Zhu and Paul, 2010; Campbell et al., 2015; Gonzales, 2015). Philip et al. found that in the *P. gingivalis* infection-induced periodontitis model, Th1 cell responses were significantly increased and promoted the secretion of the pro-inflammatory factors IFN-γ, IL-1α and IL-1β; activated Th1 cells also highly express receptor activator for nuclear factor-κB ligand (RANKL) and induce osteoclastogenesis, thus causing a widespread inflammatory response and alveolar bone destruction in periodontal tissue (Stashenko et al., 2007). Unlike Th1 cells, Th17 cells mainly secrete the pro-inflammatory factors IL-6, IL-17 and IL-23 to regulate the development of periodontitis, among which IL-17 can promote periodontal connective tissue and alveolar bone destruction by regulating the expression levels of prostaglandin E2 (PGE2), matrix metalloproteinases (MMPs) and RANKL (Cheng et al., 2014; Dutzan and Abusleme, 2019; Huang et al., 2021; Kini et al., 2022). In contrast, Treg cells play a role in reducing the inflammatory response mainly through the secretion of anti-inflammatory factors IL-10, IL-35 and transforming growth factor β (TGF-β) (Alvarez et al., 2018; Zhang et al., 2021b). Zheng et al. found a Th17/Treg imbalance in patients with chronic periodontitis, in which the Th17 ratio was upregulated and the Treg ratio was downregulated, and the expression level of the pro-inflammatory factor IL-17 was significantly increased while the expression of the anti-inflammatory factor IL-10 was decreased, and this imbalance ultimately led to the resorption of alveolar bone (Zheng et al., 2019). Furthermore, recent studies have found that Tregs (CD25/Foxp3 double-positive cells) may lose Foxp3 expression and convert to exFoxP3 Th17 cells in periodontitis, and decreased Tregs activity reduces the synthesis of TGF-β and IL-10, while these exFoxp3 Th17 cells express high levels of RANKL and IL-17; in contrast, when antibiotics were used to reduce the conversion of Treg cells, the Th17/Treg cell imbalance in periodontitis in mice was significantly inhibited and RANKL expression was suppressed, resulting in improved bone resorption (Deng et al., 2021).

In both the innate and adaptive immune responses, macrophages, neutrophils and T cells are important target cells for bacterial invasion of the host, and pathogen molecules can be recognized by surface receptors (e.g., toll-like receptors, TLR) of these cells, initiating intracellular signaling pathways that lead to the release of various inflammatory and chemotactic factors (Kawai and Akira, 2007; Oberg et al., 2011; Fitzgerald and Kagan, 2020), such as macrophage colony-stimulating factor (M-CSF) and RANKL, both of which are key factors in the regulation of osteoclast activity (Cekici et al., 2013). For example, *P. gingivalis* can activate TLR2 and TLR4 in a variety of cell types, and TLR2 activation can upregulate the expression of M-CSF, thus promoting osteoclast activation (Karlis et al., 2020); meanwhile, bacterial LPS can induce IL-1β production through the TLR pathway to upregulate RANKL, and binding of RANKL to its receptor RANK triggers nuclear factor κB (NF-ĸB) signaling pathway, which induces bone resorption (Cekici et al., 2013; Thummuri et al., 2015; Tsukasaki, 2020). NF-ĸB is a specific transcription factor that plays a major role in the inflammatory response. It is a key transcription factor for intrinsic immune cells (e.g., macrophages) and also plays a role in adaptive immunity by regulating TCR signaling to promote differentiation of Th1 and Th17 cells, and its activation induces the production of pro-inflammatory cytokines such as IL-1, IL-6, IL-12, IL-17 and TNF-α (Liu et al., 2017; Venugopal et al., 2018; Mooney and Sahingur, 2020). In addition, myeloid differentiation factor 88 (MyD88), a key junction molecule in the TLR signaling pathway, binds to the activated TLR/IL-1R (TIR) structural domain, which in turn transmits signals and releases a series of pro-inflammatory factors (Kim et al., 2019; Chen et al., 2020); on this basis, Madeira et al. showed that bacterial LPS can activate the TLR4 pathway through MyD88 signaling to stimulate RANKL expression and induce osteoclast activation; while MyD88 knockout mice reduced alveolar bone resorption by downregulating TNF-α levels (TNF-α increases the expression of M-CSF and RANKL) levels (Madeira et al., 2013). George et al. found that *P. gingivalis* also activates complement C5a receptor-1 (C5aR-1) and TLR2 in neutrophils and disarms the host protective TLR2-MyD88 pathway *via* proteasomal degradation of MyD88, while the close association between TLR2 and C5aR activates the phosphatidylinositol 3 kinase (TLR2-Mal-PI3K) pathway, which further prevents phagocytosis by neutrophils, facilitates the invasion of other susceptible bacteria, and stimulates the production of inflammatory cytokines, thereby promoting an inflammatory response (Maekawa et al., 2014). Besides, MMP can also be involved in the process of tissue destruction in periodontitis by degrading the extracellular matrix. Luchian et al. showed that MMP-8 and MMP-9 are the main metalloproteinases participating in periodontal tissue destruction; MMP-8 activation promotes neutrophil migration into the gingival sulcus, leading to extensive destruction of periodontal tissue; while MMP-9 regulates IL-1, IL-6 and IL-8, engaging in the breakdown of proteins in connective tissue and playing a key role in osteoclast-induced bone resorption (Luchian et al., 2022). The high expression of MMP-8 and MMP-9 in chronic periodontitis accurately reflects the patient’s condition and therefore can be used as a biomarker for the diagnosis of periodontitis.

The pathogenesis of IBD is similar to that of periodontitis and is also regulated by a combination of intrinsic and adaptive immunity. Neutrophils and macrophages play an important role in the intrinsic immune response in IBD. Neutrophil activation is followed by the disruption of the intestinal epithelial barrier through the production of high levels of reactive oxygen species (ROS) and the release of proteases, pro-inflammatory cytokines and chemokines, such as IL-8, TNF-α and leukotriene B4 (LTB4)(Wéra et al., 2016; Zhou and Liu, 2017; Kvedaraite, 2021); the number of macrophages in the intestinal mucosa in IBD patients is increased, and the intestinal epithelium damage is aggravated by producing a large number of pro-inflammatory and chemokines, such as TNF-α, IL-1β, IL-12 and IL-23, and enhancing the Th1 and Th17 cell responses (Bain and Schridde, 2018; Na et al., 2019; Pan et al., 2022). CD4^+^ T cell-mediated adaptive immunity also plays a crucial role in the development of IBD, and Neurath et al. found that CD is characterized by Th1 immune responses, with Th1 increasing the secretion of the pro-inflammatory factors IFN-γ and IL-2, while UC is a Th2-mediated disease that produces excessive amounts of IL-5 and IL-13 (Neurath et al., 2002). In addition to secreting inhibitory cytokines, Treg cells influence the progression of IBD through cytokine deprivation-induced apoptosis and IL-2. CD4^+^CD25^+^Foxp3^+^Treg cells express all three components of the high-affinity IL-2 receptor (IL-2R): CD25, CD122 and CD132, which may compete with responder Foxp3^-^T cells for IL-2, consume it and inhibit the proliferation of Foxp3^-^T cells by suppressing the induction of IL-2 mRNA (and mRNA for other effector cytokines) in responder Foxp3^-^T cells (Shevach, 2009). The constitutive expression of the high-affinity IL-2 receptor containing CD25 allows Treg cells to sustain cytokine uptake in their environment, and Pandiyan et al. showed that in the presence of the pro-apoptotic protein Bim, Treg cells in mouse models of IBD can induce apoptosis of effector CD4^+^ T cells. The apoptotic pathway triggered is typical of cytokine deprivation, being mediated by inactivation of the anti-apoptotic phosphatidylinositol-3-OH kinase–dependent Akt kinase–Bcl-2 antagonist of cell death pathway and being dependent on the death-promoting protein Bim (Pandiyan et al., 2007); furthermore, IL-2 is not only important for the regulation of Treg cell homeostasis *in vivo*, but also has a potent inhibitory function. Zhou et al. found that group-3 innate lymphoid cells (ILC3s) in the intestine are the main cellular source of IL-2, and that CD patients showed significant depletion of IL-2 in the small intestine and a significant decrease in Treg levels (Zhou et al., 2019). Clinical studies have demonstrated that low doses of IL-2 can mediate immunomodulation and specifically expand and activate the Treg cell population, thus serving as a potential treatment strategy for patients with IBD (Klatzmann and Abbas, 2015). In recent years, some studies have found that bile acid metabolites, intestinal microbes and TCR signaling intensity act synergistically to influence the balance of Th17/Treg cells, which in turn exacerbates the progression of IBD (Britton et al., 2019; Hang et al., 2019; Yan et al., 2020; Liu et al., 2020b). In particular, bile acids control Th17 cell function by regulating the activity of the characteristic transcription factor RORγt, while a decrease in the abundance of *Firmicutes* in the intestine of IBD patients leads to a decrease in secondary bile acid levels, which upregulate Th17 differentiation and induce an inflammatory response (Fitzpatrick and Jenabzadeh, 2020); Hemarajata et al. found that intestinal microbes and their bacterial products (e.g., *Escherichia coli*) can directly act on TLR and other innate immune receptors to mediate the differentiation of Th17 cells and suppress Treg cells, thereby exacerbating intestinal inflammation (Hemarajata and Versalovic, 2012).

Patients with IBD have damage to intestinal tight junction (TJ) barrier, which is manifested by increased permeability of the intestine. NF-κB is involved in regulating TJ barrier function and increasing permeability in animal models of colitis (Yu et al., 2018; Kaminsky et al., 2021; Li et al., 2022a), and studies have found that NF-κB activity is increased in patients with IBD, and inhibition of NF-κB signaling conduction improved the symptoms of colitis in mice (Lin et al., 2019). Additionally, MMP-9 inhibits cell adhesion and wound repair, which is elevated in intestinal tissues, serum and feces of IBD patients and is strongly correlated with disease activity and degree of inflammation (Meijer et al., 2007; Liu et al., 2013; de Bruyn and Ferrante, 2018), while MMP-9 deficiency attenuates intestinal inflammation in mice (Moore et al., 2011). Rana et al. demonstrated that MMP-9 can increase myosin light chain kinase (MLCK) gene and protein expression through NF-κB p65 activation, thereby inducing increased intestinal TJ permeability (Al-Sadi et al., 2021).

**Figure 3:**
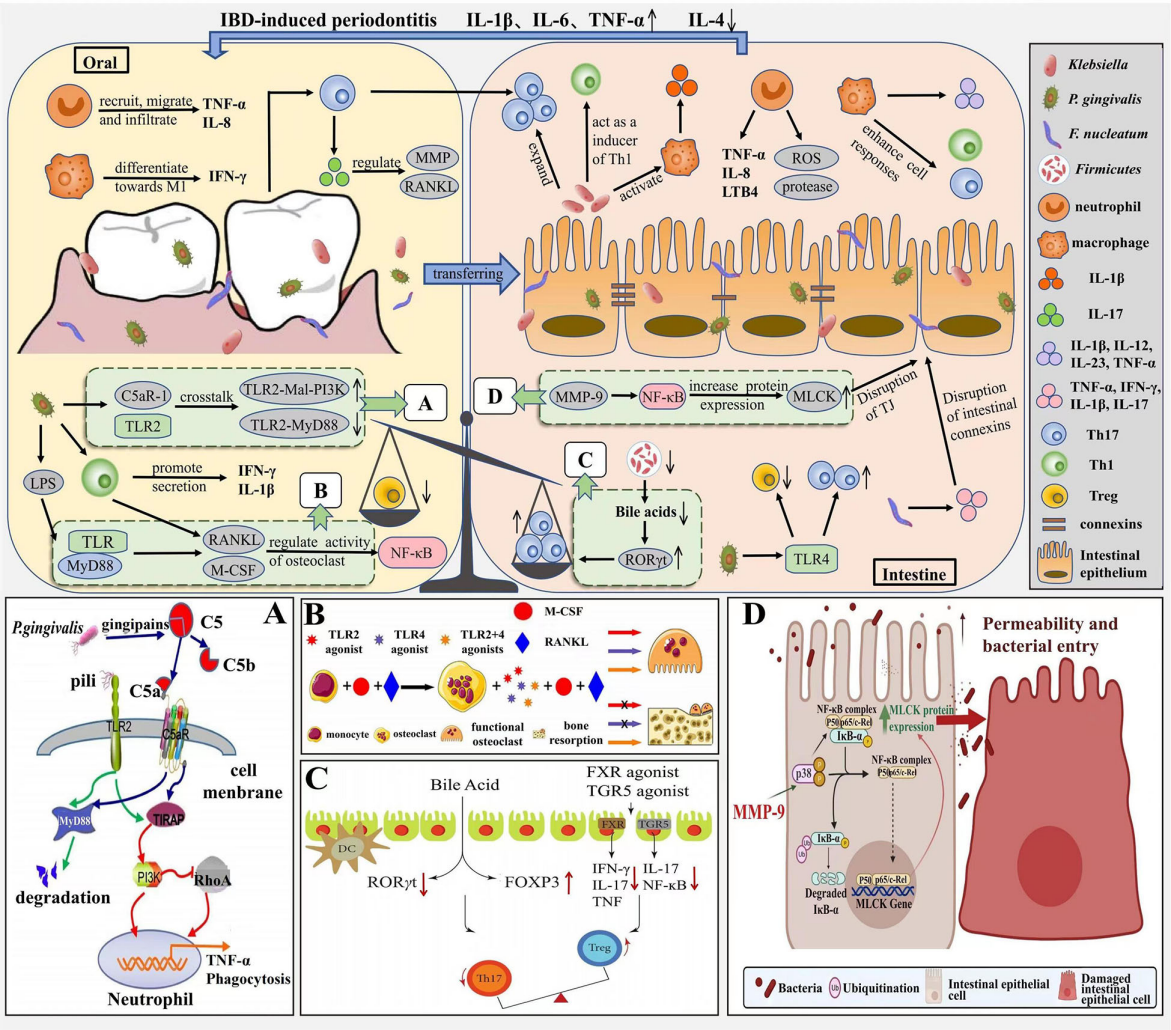
Immune mechanism correlation between periodontitis and IBD. In the pathogenesis of periodontitis, neutrophils are continuously recruited, migrate and infiltrate at the site of inflammation and secrete pro-inflammatory factors such as TNF-α and IL-8, macrophages tend to differentiate towards M1 and secrete IFN-γ. *P. gingivalis* induces IL-1β production through the TLR pathway, upregulates RANKL and M-CSF expression, and activates the NF-ĸB signaling pathway, thereby inducing bone resorption. The pathogenesis of IBD is similar to periodontitis, with neutrophils and macrophages inducing an inflammatory response through the release of virulence factors, pro-inflammatory mediators, and enhanced Th1 and Th17 cell responses; MMP-9 increases MLCK expression and induces an increase in intestinal TJ permeability through activation of NF-κB p65. Due to the oral-intestinal axis, periodontitis and IBD can be interrelated through immune mechanisms, with periodontal pathogenic bacteria ectopically colonizing the intestine, increasing intestinal Th17 and Th1 cell responses and promoting intestinal inflammation; while Th17/Treg imbalance not only disrupts the oral microbiota and exacerbates bone resorption, but also leads to intestinal ecological dysregulation. **(A)** Antibactericidal mechanisms of C5a-TLR2 cross-talk induced by *P. gingivalis*. Source (Jia et al., 2019a). **(B)** M-CSF and RANKL induce bone resorption. Source (Karlis et al., 2020). **(C)** The role of bile acid and FXR in Th17/Treg balance. Source (Yan et al., 2020). **(D)** MMP-9 causes increased intestinal permeability. Source (Al-Sadi et al., 2021).

Periodontitis and IBD can be interrelated through immune mechanisms due to the presence of the oral-gut axis (**Figure 3**). Especially, pro-inflammatory cytokines are associated with the pathogenesis of both periodontitis and IBD. IL-1β is known to influence the progression of periodontitis and IBD, and Kitamoto et al. found that *Enterobacteriaceae* isolated from the intestine were unable to induce IL-1β secretion, whereas *Enterobacteriaceae* in the oral cavity (e.g., *Klebsiella*) could induce intestinal inflammation by activating inflammatory vesicles in macrophages mediating IL-1 signaling (Kitamoto et al., 2020b). Liu et al. found that *F. nucleatum*, an important causative agent of periodontitis, increases intestinal epithelial permeability, disrupts its integrity by disrupting connexins, promotes the secretion of cytokines TNF-α, IFN-γ, IL-1β, IL-6 and IL-17 and reduces the secretion of anti-inflammatory cytokine IL-10, thereby increasing intestinal inflammation (Liu et al., 2020a). In addition, periodontitis leads to T cells, and pathogenic T cells can migrate from the oral cavity to the intestine, as periodontitis triggers the production of oral Th17 cells, which can migrate to the intestine and expand in response to ectopically colonized Klebsiella, thus promoting worsening intestinal inflammation (Kitamoto et al., 2020b); Koji’s study concluded that *Klebsiella*, after colonization of the intestine, induces Th1 cell responses by stimulating the innate immune receptor TLR4 and leads to a deficiency of the immunosuppressive factor IL-10, thereby promoting inflammatory responses in the intestine, thus demonstrating that *Klebsiella*, isolated from the oral microbiota, can act as a strong inducer of intestinal Th1 cells (Atarashi et al., 2017). The Th17/Treg ratio is closely related to the development of IBD, and Lu et al. found that *P. gingivalis* in periodontitis stimulates CD4^+^ T cell responses and increases the Th17/Treg ratio in the intestinal lamina propria, upregulates Th17-related transcription factor expression and production of the pro-inflammatory factors IL-17 and IL-6 through the TLR4 pathway, and downregulates the expression of the Treg transcription factor Foxp3 and production of the anti-inflammatory factors TGF-β and IL-10, thereby exacerbating intestinal inflammation (Jia et al., 2020). In oral and intestinal inflammation due to oral *Candida* infection, the Treg population has a multilayered protective role, one for IL-17A induction in CD4^+^ T cells and another for immunomodulation to prevent excessive inflammation. In a study by Bhaskaran et al, Foxp3 and IL-17A expression in CD4^+^ T cells was effectively induced by supplementation with SCFA, thereby increasing the frequency of Foxp3^+^ Tregs and thus improving the inflammatory response due to *Candida* infection (Bhaskaran et al., 2018).

Meanwhile, the immune response associated with IBD may also contribute to oral inflammation. Dysbiosis of the gut flora not only damages the intestinal barrier, but also disrupts the oral microbiota and exacerbates bone resorption in periodontitis through Th17/Treg imbalance. Yuan et al. found that long-term antibiotic use led to gut ecological dysbiosis, which increased periodontitis-associated pathogens in the oral cavity and decreased oral microbiota probiotics associated with periodontal health, while Th17 cell-associated pro-inflammatory cytokine (IL-17A, IL-6) expression was upregulated and Treg cell-associated cytokine (Foxp3 and IL-10) expression was decreased in periodontal tissues; in contrast, the use of fecal microbiota transplantation (FMT) not only restored the intestinal microbiota of the mice, but even reversed the Th17/Treg imbalance in periodontal tissue and alleviated periodontitis(Yuan et al., 2023). Katarzyna et al. found elevated levels of IL-1β, IL-6 and TNF by measuring salivary inflammatory markers in patients with IBD, with elevated levels of TNF-α and IL-6 being strongly associated with the development of periodontitis (Szczeklik et al., 2012); in the Figueredo team’s study, inflammation scores in gingival tissue were significantly higher in patients with active IBD (including four cytokines, IL-1β, IL-6, IL-21 and sCD40L) (Figueredo et al., 2017); nevertheless, anti-inflammatory factors such as IL-4 decrease with increasing levels of inflammation, and IL-4 levels were found to be significantly lower in the gingival sulcus of IBD patients with periodontitis (de Mello-Neto et al., 2021). This evidence suggests that IBD may be closely associated with the development of periodontitis by decreasing the immune defenses of periodontal tissues.

## 3 Summary and prospect

At present, there is a growing body of research suggesting a bidirectional association between periodontitis and IBD, with microbial and immune factors combining to influence the development of both diseases (**Table 1**). Bacteria invade host tissues and cause ecological dysbiosis that damages host tissues and induces host immune responses, leading to inflammation. Periodontitis-associated pathogenic bacteria can ectopically colonize the intestinal tract, thereby exacerbating intestinal inflammation, as evidenced by a higher prevalence of IBD in patients with periodontitis, while the inflammatory response to IBD can alter the composition of the oral microbiota, as evidenced by a higher prevalence and severity of periodontitis in patients with IBD. Among the microbial factors, we highlight the role of *Klebsiella, P. gingivalis, F. nucleatum* and *Candida* through the oral-gut axis in both diseases and the modulation of inflammation by microbial metabolites such as short-chain fatty acids; while among the immune mechanisms, the role of cytokines and the Th17/Treg balance contribute to the bidirectional effect between periodontitis and IBD.

**Table 1:** Key factors in the oral-intestinal axis (both microbiological and immunological).

periodontitis	Microbiological factors	*P.gingivalis* *F.nucleatum* *Tannerella forsythia* *Treponema denticola* metabolites (SCFAs)
	Immune factors	neutrophils macrophages Th17/Treg imbalance TNF-*α*, IFN-γ, TGF-β IL-1, IL-6, IL-8, IL-12, IL-17, IL-23 PGE2 MMPs M-CSF, RANKL NF-κB
IBD	Microbiological factors	*Firmicutes* *Bacteroidetes* *Proteobacteria* *Actinobacteria* *Faecalibacterium prausnitzii* metabolites(BSH, butyrate, TMAO)
	Immune factors	Neutrophils Macrophages Th17/Treg imbalance TNF-*α* LTB4 IL-1β, IL-5, IL-8, IL-12, IL-23, IL-13 NF-κB MMP-9
Acting simultaneously in both diseases *via* the oral-gut axis	Microbiological factors	*Klebsiella* *Proteus* *Candida* *P. gingivalis* *F. nucleatum* metabolites(Ile,TMAO)
	Immune factors	Th17/Treg imbalance TNF-*α* IFN-γ IL-1β, IL-6, IL-17

In this article, we mentioned that previous research with probiotic supplementation, dietary control and exosomes can treat one disease while benefiting the control of the other. In addition to this, recent studies have found that related drugs exert important anti-inflammatory effects in both the intestinal and oral cavity *via* the oral-gut axis. In addition to this, recent studies have found that related drugs exert important anti-inflammatory effects in both the intestinal and oral cavity *via* the oral-gut axis. Berberine has been shown to be widely used in the treatment of gastrointestinal disorders caused by microbial infections. Jia et al. demonstrated that berberine increased butyrate production in the intestinal microbiota, effectively restored the intestinal barrier and significantly inhibited IL-17-related systemic and local immune responses in ovariectomized (OVX)-periodontitis rats, resulting in improved periodontal bone resorption (Jia et al., 2019b); moreover, quercetin supplementation was effective in restoring intestinal microbiota, enhancing butyrate production in the gut and significantly improving intestinal ecological dysbiosis in mice treated with antibiotics (Shi et al., 2020), and Mooney et al. found that oral quercetin helped restore periodontal tissue homeostasis and improved periodontitis by modulating the inflammatory response and oral microbial composition (Mooney et al., 2021). These studies open up new ideas for the future treatment of periodontitis, that is, to prevent and control local inflammation in the oral cavity by modulating the intestinal flora or immune response while improving intestinal inflammation or systemic inflammation with medication.

While numerous studies have demonstrated a close link and bidirectional effects between periodontitis and IBD, there are still some limitations. Periodontitis is an inflammatory disease caused by dental plaque and is usually closely related to oral hygiene, patients with chronic periodontitis are usually accompanied by poor oral hygiene, however, studies have pointed to an inverse correlation between poor oral health and IBD. People with IBD brush their teeth, floss and breath fresheners more frequently, and those with CD have less plaque. excessive oral hygiene may lead to dysbiosis in bacterial colonization, dysregulated innate immune response, and promote inflammatory processes; conversely, poor oral health may help induce immune tolerance and suppress overreactive inflammation, thereby reducing the risk of immune-mediated diseases such as IBD (Yin et al., 2017). In addition, there are some confounding factors such as gender, age and genetics for the study of the association between periodontitis and IBD. Chemokines act in the recruitment of neutrophils and T lymphocytes to the epithelium, which may trigger or promote inflammatory responses, and one study showed enhanced production of chemokines CXCL-8, CXCL-9 and CXCL-10 in oral buccal epithelial cells only in children with CD compared to healthy controls and adults with CD (Damen et al., 2006). Periodontitis and IBD are both chronic inflammatory conditions based on a complex interaction of genetic, environmental, microbial and immune factors, and both share similar mechanisms in terms of bacterial action and specific host responses, but there are also different factors. Smoking, for example, is considered a risk factor for both periodontitis and CD. However, its role in UC is unclear, and some studies have even reported a protective effect of smoking attributed to this condition (Indriolo et al., 2011).

In general, the relationship between periodontitis and IBD is a two-way influence and even forms a vicious circle. In this paper, we elaborate the mechanism of the intrinsic connection between the two diseases through both microbiological and immunological mechanisms, which provides us with directions for the future treatment of periodontitis. In terms of microbiology, we can evaluate the oral and intestinal microenvironment to find the common pathogens or microbial metabolites of both diseases, which can be used as biomarkers for diagnosis and even prevention of both diseases, and through drug treatment, while regulating the intestinal flora, *via* the oral-gut axis can also play a key role in the treatment of periodontitis; additionally, regulating Th17/Treg balance or inhibiting the related inflammatory signaling pathways through immune means can be a common therapeutic target for both diseases. All these deserve further thought and research.

## Author contributions

TZ and WX wrote the manuscript; QW, CJ, and YC sorted literatures; HL, YS and LA revised the review. All authors contributed to the article and approved the submitted version.
